# Multiple sclerosis as an under-recognized cause of acute vertigo: clinical and pathophysiologic insights

**DOI:** 10.3389/fneur.2026.1888822

**Published:** 2026-08-26

**Authors:** Evangelos Anagnostou, Maria Kouvli

**Affiliations:** Department of Neurology, Eginition Hospital, National and Kapodistrian University of Athens, Athens, Greece

**Keywords:** central positional nystagmus, eye movements, multiple sclerosis, vertigo, vestibular

## Abstract

Multiple sclerosis (MS) is an autoimmune disorder that frequently presents with acute neurological symptoms during relapsing–remitting phases. While motor, sensory, and visual deficits are widely recognized manifestations of relapses, vestibular symptoms such as acute vertigo, dizziness, and imbalance are comparatively under-recognized and often pose diagnostic challenges. This review synthesizes foundational and recent evidence regarding MS-related acute vestibular syndrome and its central pathophysiologic mechanisms. Infratentorial demyelinating plaques, most frequently localized within the pons and the middle cerebellar peduncles, disrupt large fiber systems and give rise to distinct central oculomotor signs. We highlight highly characteristic ocular motor features—including internuclear ophthalmoplegia, wall-eyed bilateral INO, and acquired pendular nystagmus—that serve as crucial topodiagnostic clues. Furthermore, we address clinical mimics, distinguishing central positional nystagmus and vertigo from benign paroxysmal positional vertigo, the latter being highly prevalent and treatable via bedside maneuvers in MS populations. Crucially, this paper examines the limitations of bedside tools like the HINTS exam, which identify central pathology but fail to differentiate ischemic stroke from acute demyelination. In the emergency setting, integrating advanced neuroimaging—such as perfusion MRI, the central vein sign, and paramagnetic rim lesions—with detailed ocular motor evaluation is vital. Lastly, we explore atypical fascicular lesions mimicking peripheral vestibulopathy and hypothesize how delayed, desynchronized axonal conduction in MS may impair the precise timing of covert saccades required for vestibular recovery, warranting future cohort investigations.

## Introduction

Multiple sclerosis (MS) is a chronic inflammatory demyelinating disease of the central nervous system driven by complex interactions between cellular and humoral immune responses, as evidenced by the success of B-cell-targeted therapies (1). It is historically viewed as a two-stage disease, with an initial inflammatory stage responsible for relapsing–remitting disease and a later appearing neurodegenerative stage characterized by non-relapsing progression termed secondary progressive MS. In a minority of patients, the disease is primary progressive from the time of onset (2).

A clinical attack, which characterizes the relapsing–remitting form of multiple sclerosis (RRMS), often prompts patients to present to the emergency department because of the acute onset of neurological symptoms. Common manifestations of a relapse include motor and/or sensory deficits affecting the limbs or face, monocular visual impairment, and bladder dysfunction. A subset of patients presents with vertigo, dizziness, imbalance, and a range of acute oculomotor deficits. The underlying cause may be either a newly formed demyelinating plaque within central vestibular structures—most commonly located in the brainstem or cerebellum—or a benign peripheral vestibular disorder unrelated to MS. Over the past two decades, clinical research on central causes of acute vertigo has focused predominantly on cerebrovascular disease, particularly ischemic stroke, yielding substantial insights into central vestibular networks and their neurological topodiagnostic relevance. In this review, we integrate evidence from both foundational and recent studies to provide a comprehensive overview of the comparatively neglected topic of vertigo in RRMS.

## Ocular motor and vestibular manifestations of multiple sclerosis in dizzy patients

In principle, a relapse of MS may present with a broad spectrum of neurological symptoms, depending primarily on the anatomical location and extent of a newly developing inflammatory demyelinating lesion within the central nervous system. Because MS lesions may affect virtually any region of the brain or spinal cord, clinical manifestations are highly variable and often reflect the functional systems involved. Among these manifestations, vertigo and dizziness are common subjective complaints, particularly when inflammatory activity involves structures responsible for vestibular processing and ocular motor control.

Vertigo and dizziness are frequently accompanied by a variety of oculomotor abnormalities, reflecting disruption of the complex neural networks that coordinate eye movements and maintain visual stability. Accordingly, newly formed MS plaques located in the brainstem or cerebellum may manifest with clinical signs such as strabismus, different forms of spontaneous, gaze-evoked, or positional nystagmus, impaired smooth pursuit eye movements, saccadic dysmetria, vergence dysfunction, and fixation intrusions. These disturbances arise from involvement of pathways integrating vestibular, cerebellar, and brainstem ocular motor nuclei, which are particularly vulnerable to demyelinating processes. However, the majority of these ocular motor disorders are nonspecific. Similar findings may occur in a wide range of acute or subacute neurological conditions, including ischemic or hemorrhagic stroke, inflammatory disorders such as rhombencephalitis, or other structural lesions affecting the posterior fossa. Consequently, these signs alone cannot be regarded as pathognomonic for MS and must always be interpreted within the broader clinical and radiological context.

In contrast, certain oculomotor abnormalities occur preferentially in central nervous system demyelination and are therefore considered highly characteristic of MS. Importantly, many of these distinctive eye-movement abnormalities are closely associated with symptoms of dizziness and/or vertigo, reflecting shared involvement of vestibulo-ocular pathways. Recognition of these specific clinical signs may therefore provide valuable diagnostic clues and help differentiate MS relapses from other neurological disorders presenting with acute vestibular symptoms.

**Figure 1:**
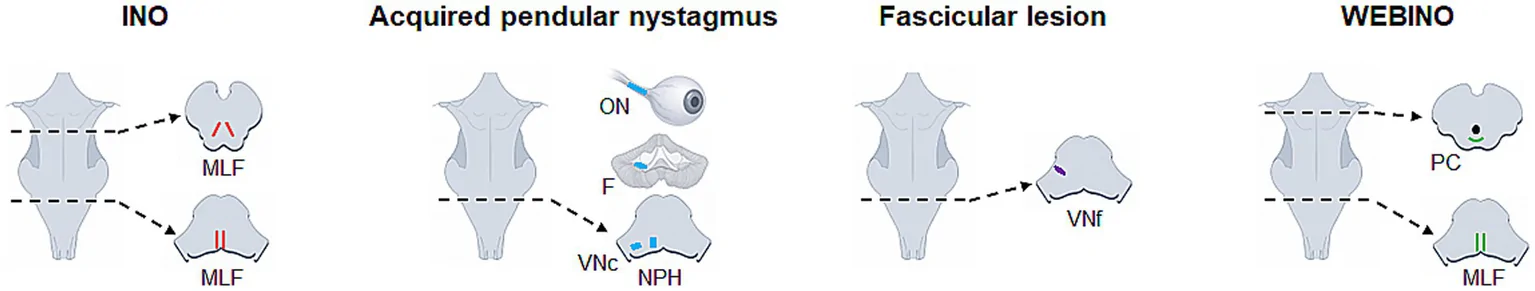
Schematic representation of putative lesions underlying oculomotor and vestibular syndromes characteristic of MS. INO may result from unilateral or bilateral lesions of the MLF anywhere along its course within the brainstem. Acquired pendular nystagmus may arise from combined lesions affecting the oculomotor neural integrator—within the brainstem (e.g., NPH and vestibular nucleus VNc) and/or its cerebellar network components (flocculus)—together with an afferent visual deficit, such as optic neuritis. A pseudo-vestibular neuritis may result from a fascicular lesion at the vestibular nerve entry zone in the brainstem. WEBINO may occur due to bilateral MLF lesions combined with involvement of vergence-related structures in the midbrain. F, cerebellar flocculus; INO, internuclear ophthalmoplegia; MLF, medial longitudinal fasciculus; NPH, nucleus prepositus hypoglossi; ON, optic nerve; PC, posterior commissure; VNc, vestibular nucleus; VNf, vestibular nerve fascicle; WEBINO, wall-eyed bilateral internuclear ophthalmoplegia.

In the following, we elaborate on four such characteristic abnormalities (Figure 1):

1. Internuclear ophthalmoplegia (INO)
2. Acquired pendular nystagmus
3. Vestibular nerve root-entry-zone lesion
4. Wall-eyed bilateral internuclear ophthalmoplegia (WEBINO)

### Internuclear ophthalmoplegia (INO)

INO is a distinctive ocular motor disorder caused by lesions of the medial longitudinal fasciculus (MLF), a paired brainstem pathway that coordinates conjugate eye movements by linking the abducens and oculomotor nuclei (3, 4). Disruption of this pathway impairs horizontal gaze coordination, producing the characteristic clinical triad of slowed or limited adduction of the eye ipsilateral to the lesion, abducting nystagmus of the contralateral eye, and varying preservation of convergence (5, 6). Patients commonly present with horizontal diplopia or oscillopsia, although symptoms may be mild despite clear examination findings. The severity of INO ranges from subtle adduction lag detectable only during large saccades to complete failure of adduction beyond the midline. Dissociation of convergence, when present, is a useful diagnostic feature distinguishing true INO from mimicking conditions such as myasthenia gravis or isolated medial rectus weakness. Because the MLF lies close to multiple brainstem structures, INO is frequently accompanied by additional neurological signs, and combined syndromes such as one-and-a-half syndrome or other “INO-plus” variants may occur. A variety of pathological processes can affect the MLF, most commonly ischemic infarction and demyelination. Importantly, the laterality of INO often provides an etiological clue. Large clinical series demonstrate that ischemic lesions are typically unilateral, whereas demyelinating disease more often produces bilateral involvement (7). Consequently, bilateral INO is particularly characteristic of multiple sclerosis and represents one of the most localizing ocular motor signs of this condition, especially in younger patients. Bilateral cases may also show additional features such as vertical eye movement abnormalities or, rarely, wall-eyed bilateral INO with exotropia. Recognition of INO therefore has substantial diagnostic value, as careful bedside examination of eye movements can accurately localize a brainstem lesion and suggest the underlying pathology. Overall, INO illustrates how disruption of a single integrative brainstem tract can produce a highly specific pattern of ocular motor dysfunction that remains clinically important for diagnosing demyelinating disease, particularly MS.

### Acquired pendular nystagmus

Acquired pendular nystagmus (APN) is a characteristic ocular motor oscillation that may be observed in patients with MS (8). It consists of quasi-sinusoidal, conjugate eye movements without a clear fast or slow phase, typically occurring at low frequency and variable amplitude. Current pathophysiological models implicate instability of the brainstem–cerebellar gaze-holding and visual feedback networks. In particular, lesions involving the brainstem neural integrator and its cerebellar connections can lead to oscillatory instability within the ocular motor control loop. In MS, APN is frequently considered a “double-crash” syndrome, in which a central lesion affecting the ocular motor network interacts with an afferent visual disturbance, most commonly prior optic neuritis. Demyelination of the optic nerve introduces delayed and degraded visual feedback, which further destabilizes the ocular motor control system and promotes sustained oscillations. Clinically, APN in MS is often associated with dizziness, oscillopsia and reduced visual acuity and may be binocular or dissociated between the eyes (9, 10).

### Vestibular nerve root-entry-zone lesion

The clinical neurologist can readily recognize a central vestibular lesion (as opposed to a peripheral one) caused by involvement of the vestibular nucleus. This is possible because, in addition to symptoms that mimic a peripheral lesion—such as an impaired ipsilesional head-impulse test and spontaneous nystagmus—central signs are also present, including gaze-evoked nystagmus and impaired smooth pursuit. These findings reflect the role of the vestibular nucleus both as a neural integrator and as a component of the smooth-pursuit control pathway. Infarction is the most common cause of such lesions (11, 12).

In contrast, a demyelinating plaque, which preferentially affects fibers rather than neuronal perikarya, may be strategically located at the entry zone of the vestibular nerve. This produces what is referred to as a fascicular lesion. Such a lesion can more or less completely mimic a peripheral vestibulopathy and has indeed been described in the course of multiple sclerosis (13–16).

### Wall-eyed bilateral internuclear ophthalmoplegia (WEBINO)

WEBINO is an uncommon but distinctive ocular motility disorder representing a bilateral variant of internuclear ophthalmoplegia caused by lesions affecting both medial longitudinal fasciculi (MLF) within the brainstem (17). The syndrome is characterized by a unique constellation of findings including large-angle exotropia in primary gaze, bilateral impairment of adduction, and abducting nystagmus of each eye, often accompanied by vertical gaze abnormalities and vestibulo-ocular reflex dysfunction. These clinical manifestations arise from disruption of neural pathways responsible for coordinating horizontal conjugate eye movements and vestibular eye movements, highlighting the central role of the MLF in integrating ocular motor control. Although several mechanisms have been proposed to explain the prominent exotropia seen in WEBINO, its precise anatomical basis remains debated, with current evidence suggesting complex involvement of brainstem pathways rather than isolated oculomotor nuclear damage.

A wide range of pathological processes may produce bilateral MLF lesions, however, demyelinating disease—particularly multiple sclerosis—emerges as a key underlying etiology (18–20). Clinical series consistently demonstrate that bilateral forms of internuclear ophthalmoplegia are more frequently associated with multiple sclerosis than with ischemic disease, making WEBINO a highly suggestive ocular motor manifestation of demyelination, especially in younger patients. The course of WEBINO varies according to the underlying disorder, and spontaneous improvement may occur, particularly in multiple sclerosis due to remyelination during relapsing–remitting phases. Management is primarily directed toward alleviating persistent diplopia and may include prisms, botulinum toxin injections, or strabismus surgery in stable cases. Overall, recognition of WEBINO is clinically important because its characteristic ocular motor pattern provides valuable localization to bilateral brainstem pathways and strongly points toward multiple sclerosis as an underlying cause. Other demyelinating disorders affecting the brainstem, such as anti–aquaporin-4 antibody–positive neuromyelitis optica, may also present with WEBINO. Nevertheless, alternative causes—particularly brainstem stroke (21) and progressive supranuclear palsy (22, 23)—should be considered in the differential diagnosis.

## Vertigo in multiple sclerosis

Although central vertigo and dizziness have been extensively characterized in the context of ischemic stroke (24, 25), vestibular symptoms also represent a relatively common presenting manifestation of MS. Supplementary Table 1 (15, 26–68) summarizes data from MS cohorts, case series, and individual case reports identified in the English-language literature, describing patients presenting with vertigo and dizziness, together with the corresponding lesion localizations within the central nervous system (CNS), where available. Both single-case reports and case series have described MS-related vertigo and dizziness resulting from lesions occurring at various levels of the brainstem, the cerebellar peduncles, the cerebellar hemispheres, and, less commonly, the cerebral hemispheres.

Unfortunately, many of the reported cases were not accompanied by sufficiently detailed information on the anatomical lesion sites responsible for the vestibular symptoms. Of the 123 reported cases of MS-related vertigo and/or dizziness, only 64 allowed identification of the exact lesion location. The pons was the most frequently reported plaque location (*n* = 25), followed by the middle cerebellar peduncle (*n* = 12), the medulla (*n* = 10), the inferior cerebellar peduncle (*n* = 6), the superior cerebellar peduncle and cerebellar hemisphere (*n* = 4 each), and the midbrain (*n* = 3).

### Benign paroxysmal positional vertigo (BPPV)

BPPV is the most common vertigo syndrome, with a lifetime prevalence of posterior canal BPPV—the most frequently encountered type—of 2.4% (69). Therefore, in addition to the central causes of vertigo resulting from demyelinating central vestibular lesions, BPPV remains a frequent cause of vertigo in MS patients (15). Indeed, some case series suggest that BPPV is the most common cause of acute vertigo in MS, followed by newly appearing demyelinating infratentorial plaques (70). From a practical standpoint, recognizing BPPV in the emergency room and distinguishing it from an MS exacerbation is critically important to avoid unnecessary costs, diagnostic tests, and inappropriate treatments. This is especially relevant because BPPV in MS can be treated on-site using liberatory maneuvers, similar to BPPV in the general population (71, 72).

### Central positional nystagmus and vertigo (CPNV)

Although BPPV is likely the most frequent vertigo syndrome in MS, clinicians should maintain a high level of awareness for its principal mimic, CPNV. CPNV is characterized by positional nystagmus whose plane of beat does not correspond to the stimulated semicircular canal (e.g., downbeat nystagmus during the Dix–Hallpike maneuver), and by the absence of onset latency, lack of fatigability, and the presence of associated central oculomotor signs (including gaze-evoked nystagmus, impaired smooth pursuit, and saccadic dysmetria) (73). However, with the exception of the latter feature, the remaining characteristics may also, in principle, be observed in rarer variants of BPPV beyond the typical canalithiasis of the posterior semicircular canal. The pathophysiology of CPNV remains incompletely understood. It likely arises from lesions in the cerebellar, the brainstem or their connection, disrupting vestibular integration, gaze-holding networks, and otolith–canal signaling, producing gravity-dependent ocular motor imbalance (74). Numerous cases of CPNV secondary to cerebellar, and less frequently pontine, demyelinating lesions have been described (75–77). The cerebellar peduncles, which are major white matter tracts and therefore a common predilection site for MS lesions, have frequently been reported to harbor plaques (sometimes only a few millimeters in size and thus requiring high-resolution neuroimaging for detection) associated with CPNV (40, 42, 60, 63, 78).

## Discussion

As inferred from the cases described thus far, MS-related acute vestibular syndrome (AVS) is most frequently encountered in pontine lesions. Lesions affecting the cerebellar peduncles are also highly common, with the middle cerebellar peduncle being the most frequent site. This finding is unsurprising given the demyelinating nature of MS, which preferentially affects large fiber systems. The compiled data demonstrate that acute vertigo and dizziness can manifest as direct expressions of an MS exacerbation, occurring in various forms and often accompanied by a diverse array of central oculomotor signs. Unfortunately, only a minority, if any, of reports provided information on oculomotor and vestibular examination findings, imaging protocols, auditory symptoms, or other parameters that are currently considered standard in vestibular neurology.

The majority of systematic research on AVS has historically focused on ischemic stroke, resulting in a disproportionate emphasis on cerebrovascular causes within the diagnostic literature. This imbalance has fostered the widespread but incorrect belief that bedside tools developed in this context—particularly the HINTS examination—can reliably differentiate stroke from peripheral vestibular disorders in all patients presenting with dizziness. However, HINTS is a syndrome-specific examination designed for continuous AVS with spontaneous nystagmus and should not be applied indiscriminately to patients with other forms of dizziness, including episodic vestibular symptoms or positional vertigo.

In reality, these tools are designed to indicate the presence of a central vestibular disorder; they cannot reliably differentiate a cerebrovascular event from an acute MS demyelinating exacerbation. This limitation presents a critical clinical challenge. It can either delay targeted MS interventions, such as intravenous corticosteroid therapy, or, perhaps more concerningly, lead to the inappropriate administration of high-dose steroids to a patient experiencing an active ischemic stroke (for instance, an ischemic event occurring in a patient with a known history of MS). Consequently, advanced neuroimaging techniques are becoming increasingly vital in the emergency setting. Imaging modalities optimized for detecting early brain ischemia, including perfusion imaging (79, 80), utilized alongside newer techniques for identifying active demyelination—such as the visualization of the central vein sign and paramagnetic rim lesions (81, 82)—may assist clinicians in accurately distinguishing between these two central pathologies.

Despite these imaging advances, the initial diagnostic approach remains anchored in the detailed neurological examination. Maintaining a high index of suspicion for oculomotor signs that are highly characteristic of—though not pathognomonic for—MS, such as INO, acquired pendular nystagmus, and the WEBINO syndrome, should immediately raise concern for demyelination as the underlying etiology of an AVS.

Another noteworthy observation from this literature review is the existence of reports describing MS-related vertigo and associated oculomotor abnormalities in the complete absence of infratentorial (brainstem or cerebellar) demyelinating lesions on standard brain MRI, where only periventricular or cortical lesions were identified within the cerebral hemispheres (26, 35, 58, 59, 61, 62, 66, 67). We interpret these clinicoradiological associations with caution. Instead, we hypothesize that suboptimal imaging resolution of the brainstem and cerebellum likely failed to detect small, strategically located lesions responsible for the vestibular symptoms. Indeed, previous literature emphasizes that high-resolution, thin-slice MRI of infratentorial structures is frequently required to visualize subtle demyelinating plaques in selected cases (40, 42).

A final point of interest emerging from the published literature concerns AVS resulting from demyelinating plaques located precisely at the root entry zone of the vestibular nerve within the brainstem. On clinical and neurophysiological grounds, these patients present with a syndrome that is virtually indistinguishable from an acute unilateral vestibulopathy (AUV) of peripheral origin. While uncommon, these cases warrant dedicated study regarding their recovery trajectories compared to typical peripheral AUV patients. In peripheral AUV, it is postulated that a primary mechanism of functional recovery, alongside the gradual restoration of vestibular-ocular reflex gain, is the behavioral deployment of covert saccades. More specifically, recent evidence suggests that recovery correlates not merely with the presence of covert saccades, but with their accurate, tightly timed placement during head rotation (83, 84). If this postulation holds true, the central MS mimic of a peripheral vestibulopathy may display significantly greater inconsistency in covert saccade timing. Because the demyelination process renders axonal conduction along central vestibular pathways slower and more desynchronized, precise temporal programming may be disrupted. This hypothesis remains to be formally evaluated in a carefully selected prospective cohort.

## Conclusion

Acute vertigo and dizziness represent critical, under-recognized manifestations of multiple sclerosis relapses that demand precise clinical differentiation from both peripheral vestibulopathies and acute ischemic stroke. While bedside diagnostic tools like the HINTS examination are invaluable for identifying a central vestibular origin, they remain incapable of distinguishing a cerebrovascular event from an inflammatory demyelinating exacerbation. Consequently, relying solely on these clinical protocols introduces a profound therapeutic risk, potentially delaying necessary corticosteroid therapy for MS or prompting inappropriate steroid administration in stroke patients. Optimizing emergency triage requires a multi-faceted approach that bridges meticulous neurological examination and advanced neuroradiology. Clinicians must maintain a high index of suspicion for characteristic oculomotor markers, such as bilateral INO, WEBINO, and acquired pendular nystagmus, while actively deploying thin-slice infratentorial MRI alongside emerging imaging metrics like the central vein sign and paramagnetic rim lesions. Nevertheless, in the emergency vertigo setting, the practical availability and established clinical utility of these emerging MS-related imaging methods remain limited, and their diagnostic value awaits confirmation in prospective studies. Furthermore, unusual demyelinating lesions at the vestibular nerve root entry zone offer a unique pathophysiological window; investigating how central desynchronization affects the temporal execution of covert saccades could deeply enrich our understanding of vestibular recovery networks and guide targeted rehabilitative strategies in neuroinflammatory disease.
