# Supplementary material for: Multiple sclerosis as an under-recognized cause of acute vertigo: clinical and pathophysiologic insights
Source: Front Neurol. 2026 Aug 26;17:1888822. doi: 10.3389/fneur.2026.1888822 (PMC13555602; doi:10.3389/fneur.2026.1888822)
Supplement: Supplementary file 1 [file Table_1.docx]

**Supplementary Table 1**. Published case series and individual case reports of multiple sclerosis presenting with vestibular symptoms. Only reports providing MR-imaging data on the presumed responsible lesion site are included. For the “Additional neurological symptoms” column, “–” indicates that the patients had no neurological symptoms other than vestibular symptoms, whereas “not reported” indicates that the article did not specify whether additional symptoms were present.

| STUDY  publication year (reference number) | STUDY TYPE | NUMBER OF PATIENTS | MS STATUS | VESTIBULAR SYNDROME PHENOTYPE | VESTIBULAR AND OCULOMOTOR SYMPTOMS | HEAD IMPULSE TEST or HINTS | ADDITIONAL NEUROLOGICAL SYMPTOMS | MRI PROTOCOL | LESION SITE | TREATMENT / OUTCOME |
| --- | --- | --- | --- | --- | --- | --- | --- | --- | --- | --- |
| 1991 (26) | Small cohort | 1 | Known MS | Chronic VS | Dizziness, prolonged onset latency for rightward saccades | Not reported | Gait unsteadiness | Not reported | Periventricular hemispheric, left corpus callosum | Not reported |
| 1992 (27) | Case report | 1 | First presentation | AVS | Vertigo | Not reported | Left facial paresthesia | T2 | left middle and inferior cerebellar peduncles, pons, midbrain | 5-day iv MP  1g/day  Slow improvement |
| 1993 (28) | Case report | 1 | First presentation | AVS | Continuous rotatory vertigo, rotatory spontaneous nystagmus to the left | Not reported | Deafness of left ear | Not reported  Gd enhancement | Medulla, pons | 7-day iv prednisolone  1g/day for 3 days  And then 500mg and 250mg for one dose each  Slow improvement |
| 1994 (29) | Case report | 1 | First presentation | AVS | Vertigo, Bruns nystagmus (high amplitude and slow nystagmus to the left, spontaneous nystagmus to the right) | Not reported | Deafness of left ear | T2  Gd enhancement | left middle and inferior cerebellar peduncles | 10-day Iv MP starting with 500mg and tapered  Clinical improvement |
| 1998 (30) | Case report | 1 | Known MS | AVS | Vertigo, horizontal-rotatory nystagmus to the right | Not reported | Not reported | T2w  T1 Gd + enhancement | Medulla, pons | high dose iv steroids  full recovery |
| 1998 (31) | Case report | 1 | First presentation | T-EVS | Central positional vertigo and nystagmus, diplopia, saccadic pursuit | Not reported | Deafness of left ear, tinnitus, discoordination | Not reported | Left cerebellar peduncle, right cerebellar hemisphere | Corticosteroids 60mg/day  improvement |
| 1999 (32) | Small cohort (retrospective) | 2 | First presentation in one case-known MS in the second case | AVS | Vertigo, impaired abduction saccades in one case, INO in the second case | Not reported | - | 1.5 Tesla  T2w | pons | Not reported |
| 2000 (33) | Case series | 1 | first presentation | AVS | Dizziness, vomiting, horizontal, vertical and rotatory nystagmus | Not reported | First case: Right hemiparesis, mild limb and truncal ataxia | T2w  T1 Gd + enhancement | Hemispheres, thalamus, medulla | Pulse therapy with MP  Improvement  8 weeks after deterioration |
| 2000 (15) | Small cohort (retrospective) | 25 | Known MS | AVS | Vertigo, Dizziness (13 patients had BPPV, 4 had other vestibular disorders not related to MS) | Not reported | Wallenberg’s syndrome in one case | Not reported | Pons (6 patients), Medulla (2 patients) | All cases with BPPV were treated with Epley maneuver and recovered  7/8 cases with MS exacerbation were treated with iv steroids  3/7 improved  4/7 fully recovered  1/8 was treated with clonazepam and fully recovered |
| 2001 (34) | Case report | 1 | First presentation | T-EVS | Oscillopsia, Vertigo,  Perverted head-shaking nystagmus, central positional downbeat nystagmus | Not reported | - | T2 | Medulla | 10-day Iv adrenocortico-trophic hormone  Fully recovered |
| 2002 (35) | Case series | 2 | Known MS in one case-  First presentation in the other case | AVS | Vertigo | Not reported | Hemiparesis in one case, paresthesia in all the extremities in the other case | Not reported | Hemispheres | 3-day iv MP 1g/day  Fully recovered |
| 2003 (36) | Case report | 1 | First presentation | AVS | Dizziness, diplopia, palsy of upward gaze, convergence-retraction nystagmus | Not reported | - | T2, Flair | Midbrain | Not reported |
| 2004 (37) | Small cohort | 1 | Not specified | AVS | Vertigo | Not reported | - | Not reported | Inferior cerebellar peduncle, medulla | Not reported |
| 2004 (38) | Case report | 1 | First presentation | AVS | Vertigo | Not reported | Gait ataxia, paraparesis | T2w | Periventricular white matter, cerebellar hemispheres | Immunosup-  ressant therapy |
| 2004 (39) | Case report | 1 | Known MS | Chronic VS | Dizzy spells, gaze-evoked nystagmus | Not reported | Monocular visual impairment (optic neuritis) | T2w | Pons, cerebellar hemispheres | 3-week 50mg/day prednisolone  improvement |
| 2004 (40) | Case series | 2 | Known MS | T-EVS | Central positional downbeat nystagmus | Negative HIT | - | T2, T1 Gd+ enhancement | Superior cerebellar peduncle | 5-day iv MP 1g/day plus 2mg/day clonazepam  improvement |
| 2007 (41) | Case report | 1 | First presentation | Unspecified dizziness | Dizziness, Trochlear nerve palsy | Not reported | - | T2 | Pons, superior and middle cerebellar peduncle | No treatment  Fully recovered |
| 2008 (42) | Case report | 1 | First presentation | T-EVS | Central positional (rotational apogeotropic) nystagmus, skew deviation | Not reported | - | 1.5mm slices, T2, T1 Gd+ enhancement | Superior cerebellar peduncle | 5-day iv MP 500mg/day  fully recovered |
| 2008 (43) | Case report | 1 | First presentation | AVS | Vertigo, left beating nystagmus | Not reported | Right facial palsy and hearing loss | 1.5 Tesla MRI,  T2w, T1 Gd+ enhancement | Middle cerebellar peduncle | 7-day iv MP 1g/day  improvement |
| 2008 (44) | Case report | 1 | First presentation | Unspecified dizziness | Dizziness, right lateral rectus palsy | Not reported | Anorexia, truncal ataxia | T2, Flair,  T1 Gd + enhancement | Periventricular white matter, infratentorial | 4-day iv MP 1g/day  improvement |
| 2009 (45) | Case report | 1 | Known MS | AVS | Vertigo, horizontal diplopia | Not reported | Right hemiparesis | T1, T2 Flair | Pons, middle cerebellar peduncle | Pulse iv MP  Cyclophosphamide 800mg/m^2^/every other day iv for 10 days  improvement |
| 2010 (46) | Small cohort | 30 | Known MS | CVS (50%), AVS and unspecified dizziness | Vertigo, Dizziness | Not reported | Various other neurological symptoms and signs (not explicitly reported) | Not reported | Infratentorial lesions were found in 60% of the patients (only brainstem in 11.1%, only cerebellar in 5.5% and both in 83.3%). Additional periventricular lesions were found in 100% | 27/30 patients had pulse steroid therapy |
| 2011 (47) | Cohort (retrospective) | 12 | 3/12 first presentation  9/12 known MS | AVS | Vertigo, dizziness, 9/12 hyperventilation induced nystagmus; spontaneous downbeat nystagmus in 3 cases, a spontaneous upbeat nystagmus in one case and a spontaneous rotatory nystagmus in one case | Not reported | Not reported | Not reported | Brainstem and/or cerebellum (without further specification) | Not reported |
| 2013 (48) | Case report | 1 | Known MS | AVS | Dizziness, oscillopsia, INO, downbeat nystagmus | Not reported | - | T2w | Pons | 5-day iv MP 1g/day  improvement |
| 2013 (49) | Small cohort (prospective) | 7 | 3/7 first presentation  4/7 known MS | AVS | Vertigo, Dizziness (various oculomotor abnormalities depending on lesion site, such as unidirectional horizontal-torsional nystagmus, gaze-evoked nystagmus, upbeat nystagmus, see-saw nystagmus, skew deviation and INO) | All patients had (+) HINTS | 2/7 had additional neurological signs not further specified | T2, T1 Gd + enhancement | 3/7 pons, 1/7 inferior cerebellar peduncle, 1/7 middle cerebellar peduncle, 1/7 medulla, 1/7 hemispheric periventricular | Steroid therapy  Improvement (all of them) |
| 2014 (50) | Case report | 1 | First presentation | AVS | Dizziness, vomiting, spontaneous left beating nystagmus with an upbeat component | abnormal HIT on the right  HINTS (+) | - | T2, T1 Gd + enhancement | Middle cerebellar peduncle, cerebellar hemisphere | Steroid treatment  improvement |
| 2014 (51) | Small cohort (retrospective) plus 1 case report | 1 | Not specified | Unspecified dizziness | Dizziness, spontaneous upbeat nystagmus | Not reported | Dysphagia | 1.5 Tesla  T2, T1 | Medulla | Steroid therapy  improvement |
| 2015 (52) | Small cohort (prospective) | 1 | Known MS | AVS | Vertigo, ipsilesional spontaneous nystagmus and contralesional ocular tilt reaction and SVV tilt | Negative HIT | - | T2w | Inferior cerebellar peduncle | Therapy not reported  Fully recovered |
| 2017 (53) | Case series | 2 | First presentation | Unspecified dizziness | Dizziness, lightheadedness | Not reported | Hemifacial numbness and right hemiparesis | T2, T2 Flair, T1 Gd+ enhancement | Midbrain, Pons and juxtacortical and  periventricular region | 5-day iv MP 1g/day and carbamazepine 200mg/day  improvement |
| 2019 (54) | Case report | 1 | Known MS | CVS | Vertigo | Not reported | Hearing impairment | T2 Flair | Right middle and inferior cerebellar peduncle, pons | 3-day steroid  Pulse  improvement |
| 2020 (55) | Case report | 1 | CIS | Unspecified dizziness | Dizziness | Not reported | Ataxia | Not reported | Cerebellar hemisphere | Iv steroids  improvement |
| 2020 (56) | Small cohort (retrospective) | 4 | Known MS | Unspecified dizziness or AVS | Dizziness, bilateral INO | Not reported | Hemiparesis | 1.5 or 3Tesla Magnet, slice thickness 4mm, T1, T1 Gd +, T2, FLAIR, GRE, SWI, MPRAGE | 3/4 pons, 1/4 midbrain | Not reported |
| 2020 (57) | Case report | 1 | First presentation | AVS | Vertigo, unidirectional left-beating horizontal-torsional nystagmus (following Alexander's law) | Positive HIT to the right | Hearing loss | T2w, T1 Gd + enhancement | Middle cerebellar peduncle, cerebellar hemisphere | Iv dexamethasone 8mg and betahistine dihydrochloride 48mg  improvement |
| 2021 (58) | Case report | 1 | First presentation | Unspecified dizziness | Dizziness | Not reported | Ataxia | T2w, Flair | Hemispheric periventricular | Not specified |
| 2021 (59) | Case report | 1 | Known MS | Unspecified dizziness | Dizziness, lightheadedness, gaze-evoked nystagmus | Not reported | fatigue | T2 | Hemispheric (frontal lobe) | Iv fluids, meclizine, promethazine |
| 2021 (60) | Case report | 1 | First presentation | T-EVS | Central positional nystagmus and vertigo | Not reported | fatigue | Not reported | Middle cerebellar peduncle, periventricular white matter | Not reported |
| 2022 (61) | Case report | 1 | First presentation | Unspecified dizziness | Dizziness | Not reported | Hemifacial weakness, dysarthria | T2 Flair | Hemispheric (frontal lobe) | Not reported |
| 2022 (62) | Case series | 1 | First presentation | Unspecified dizziness | Dizziness | Not reported | Not reported | T2w | Hemispheric subcortical and periventricular | 3-day iv MP 1g/day improvement |
| 2022 (63) | Case report | 1 | First presentation | T-EVS | Central positional vertigo and nystagmus | Not reported | - | Not specified | Middle cerebellar peduncle | 5-day iv steroids  improvement |
| 2022 (64) | Case report | 1 | Known MS | AVS | Vertigo, horizontal nystagmus | Positive HIT to the left,  Negative HINTS | Ataxia | T2w, Flair | Pons | 7-day iv steroids 1g/day  improvement |
| 2023 (65) | Case series | 2 | First presentation | AVS | Vertigo, gaze-evoked-nystagmus | v-HIT positive | Tinnitus | T2, Flair, T1 | Pons, medulla, cerebellar hemispheres | Not reported |
| 2024 (66) | Case report | 1 | First presentation | AVS | Vertigo, Dizziness, Nausea | Not reported | Head tremor | T2, Flair | Hemispheric periventricular | No treatment |
| 2025 (67) | Case report | 1 | First presentation | AVS | Vertigo | Positive HIT  Positive HINTS | Vision impairment with Uhthoff phenomenon | T2, Flair | Hemispheric periventricular | 3-day iv MP 250mg/day  improvement |
| 2025 (68) | Case report | 1 | CIS | AVS | Vertigo, Dizziness, right sixth nerve palsy | Not reported | Right facial nerve palsy | Flair | Pons | Iv MP pulse therapy  improvement |

AVS: acute vestibular syndrome, CIS: clinically isolated syndrome, CVS: chronic vestibular syndrome, MP: methylprednisolone, T-EVS: triggered episodic vestibular syndrome, VS: vestibular syndrome
